# Targeted Disruption of the JAK2/STAT3 Pathway in Combination with Systemic Administration of Paclitaxel Inhibits the Priming of Ovarian Cancer Stem Cells Leading to a Reduced Tumor Burden

**DOI:** 10.3389/fonc.2014.00075

**Published:** 2014-04-09

**Authors:** Khalid Abubaker, Rodney B. Luwor, Ruth Escalona, Orla McNally, Michael A. Quinn, Erik W. Thompson, Jock K. Findlay, Nuzhat Ahmed

**Affiliations:** ^1^Women’s Cancer Research Centre, Royal Women’s Hospital, Parkville, VIC, Australia; ^2^Department of Surgery, St Vincent Hospital, University of Melbourne, Melbourne, VIC, Australia; ^3^Department of Surgery, Royal Melbourne Hospital, University of Melbourne, Melbourne, VIC, Australia; ^4^Department of Obstetrics and Gynaecology, University of Melbourne, Melbourne, VIC, Australia; ^5^St Vincent’s Institute, Fitzroy, VIC, Australia; ^6^Prince Henry’s Institute of Medical Research, Clayton, VIC, Australia

**Keywords:** ovarian carcinoma, cancer stem cells, metastasis, chemoresistance, recurrence, JAK2/STAT3 pathway

## Abstract

Chemotherapy resistance associated with recurrent disease is the major cause of poor survival of ovarian cancer patients. We have recently demonstrated activation of the JAK2/STAT3 pathway and the enhancement of a cancer stem cell (CSC)-like phenotype in ovarian cancer cells treated *in vitro* with chemotherapeutic agents. To elucidate further these mechanisms *in vivo*, we used a two-tiered paclitaxel treatment approach in nude mice inoculated with ovarian cancer cells. In the first approach, we demonstrate that a single intraperitoneal administration of paclitaxel in mice 7 days after subcutaneous transplantation of the HEY ovarian cancer cell line resulted in a significant increase in the expression of CA125, Oct4, and CD117 in mice xenografts compared to control mice xenografts which did not receive paclitaxel. In the second approach, mice were administered once weekly with paclitaxel and/or a daily dose of the JAK2-specific inhibitor, CYT387, over 4 weeks. Mice receiving paclitaxel only demonstrated a significant decrease in tumor volume compared to control mice. At the molecular level, mouse tumors remaining after paclitaxel administration showed a significant increase in the expression of Oct4 and CD117 coinciding with a significant activation of the JAK2/STAT3 pathway compared to control tumors. The addition of CYT387 with paclitaxel resulted in the suppression of JAK2/STAT3 activation and abrogation of Oct4 and CD117 expression in mouse xenografts. This coincided with significantly smaller tumors in mice administered CYT387 in addition to paclitaxel, compared to the control group and the group of mice receiving paclitaxel only. These data suggest that the systemic administration of paclitaxel enhances Oct4- and CD117-associated CSC-like marker expression in surviving cancer cells *in vivo*, which can be suppressed by the addition of the JAK2-specific inhibitor CYT387, leading to a significantly smaller tumor burden. These novel findings have the potential for the development of CSC-targeted therapy to improve the treatment outcomes of ovarian cancer patients.

## Introduction

The gold standard for the management of ovarian cancer patients after debulking surgery is the systemic administration of platinum (cisplatin/carboplatin) and taxane-based (paclitaxel) drugs. This treatment regimen results in a significant reduction of tumor burden due to substantial cancer cell death via DNA and cytoskeletal damage response pathways (1). Most of the ovarian cancer patients (~80%) respond well to the standard treatment regimen and enjoy a short-lived period of remission with asymptomatic minimal disease. However, this asymptomatic microscopic residual disease persisting after the first line chemotherapy leads to consecutive episodes of recurrent disease and eventual death. Hence, the 5-year survival period of ovarian cancer patients is as low as ~30% (2, 3). Thus, to increase the survival rate of ovarian cancer patients, there is an urgent need to identify the mechanisms that allow residual tumor cells to overcome first line chemotherapy and propagate within the changed tumor microenvironment.

Chemoresistant tumor cells that have the ability to resist the cytotoxic effects of chemotherapy have high expression of multidrug resistance transporters, enhanced ability to repair damaged DNA, and proliferate slowly (4). Recent studies have shown these phenotypes to be commonly displayed by cancer stem cells (CSCs) (5, 6). A few recent studies have also demonstrated enrichment in CSCs and stem cell mediator pathways in chemoresistant and recurrent ovarian tumors, suggesting that CSCs and their associated pathways may be important intermediaries in the emergence of disease recurrence (7–10).

Several cell signaling pathways have been associated with self-renewal and the tumorigenic phenotype of CSCs. The Wnt, Sonic Hedgehog (Shh), and the Notch signaling pathways have been shown to be the drivers for the progression of cancers, including ovarian cancer (11–13). Another signaling pathway that is implicated in ovarian as well as other solid cancers is the signal transducer and activator of transcription protein 3 (STAT3) (14–16). In normal cells, STAT3 is transiently activated in response to specific growth factors and cytokines [interleukin-6 (IL-6), granulocyte colony stimulating factor (G-CSF), leukemia inhibitory factor (LIF), epidermal growth factor (EGF), etc]. However, in cancers, including breast, ovarian, and prostate, STAT3 is constitutively active in some cancer cells (17), and is believed to be responsible for several key points in tumor progression, starting from uncontrolled cellular proliferation to the promotion of angiogenesis and importantly facilitating resistance to apoptosis induced by conventional chemotherapy (18, 19). STAT3 is also involved in integrating the signals received from a variety of external agents such as growth factors or cytokines or genotoxic stressors and mediates the response of such agents by regulating downstream gene expression linked with cell survival and other cellular functions (20–22). Moreover, the STAT3 pathway has been shown to be a requisite for the proliferation and maintenance of glioblastoma stem cells (23), as well as rapidly cycling intestinal stem cells (24).

Recent studies have shown a link between the activation of STAT3 and CSCs. Coupling of the stem cell marker CD44 with the embryonic stem cell marker Nanog has been shown to be associated with the activation of STAT3 in ovarian cancer cells (25). The activation of STAT3 in these cells resulted in multidrug resistance gene expression and concomitant chemoresistance. Furthermore, we have previously demonstrated sustained activation of the STAT3 pathway in advanced-stage ovarian tumors and in cisplatin-treated ovarian cancer cell lines (15, 26). A recent study has shown significantly enhanced activation of STAT3 sustained by infiltrating macrophages in drug-resistant recurrent ovarian tumors compared to the matched primary tumors (27). Hence, the JAK2/STAT3 pathway is a potential target for the development of novel drugs aimed at suppressing its constitutive as well as ligand-induced activation. In the last decade, several anti-STAT3 small molecule inhibitors have shown promising potential by counteracting cancer cell-associated proliferation, inflammation, and importantly chemoresistance (17, 28). However, none of these compounds have been shown to have an effect on CSCs, which theoretically have been suggested to drive chemoresistance.

We have previously demonstrated that the *in vitro* treatment of OVCA 433 and HEY cell lines with cisplatin or paclitaxel resulted in the activation of the JAK2/STAT3 pathway (7, 26). We have also shown that intraperitoneal transplantation of chemotherapy-treated cells in nude mice resulted in a significantly higher tumor burden associated with enhanced CSC-like expression compared to control untreated cells (29). In the study reported here, we aimed to determine the effect of a novel small molecule inhibitor of the JAK2/STAT3 pathway, CYT387, in combination with systemic administration of paclitaxel and assess the molecular phenotype of the resultant xenografts. We demonstrate that irrespective of the length of paclitaxel treatment, systemic administration of paclitaxel enhanced the expression of Oct4 and CD117 in residual tumors. However, administration of CYT387 (by daily oral gavages) in combination with weekly systemic paclitaxel administration resulted in a significantly reduced tumor volume compared to control and paclitaxel alone treatment mice. These tumors displayed diminished JAK2/STAT3 activation as well as diminished Oct4 and CD117 expression compared to tumors generated during systemic administration of paclitaxel only. These novel data suggest that the inclusion of a JAK2/STAT3 inhibitor such as CYT387 with paclitaxel has the potential of reducing the tumor volume further than that achieved by using chemotherapy alone. Such observations in animal models provide “proof of concept” demonstrating the potential of CYT387 in reducing the intraperitoneal tumor burden in ovarian cancer patients further than that achieved by paclitaxel on its own. This may provide the patients with a lower or/zero incidence of tumor recurrence or longer disease-free survival period, and better quality of life so lacking following the current treatment options in these patients.

## Materials and Methods

### Cell lines

The human ovarian HEY cell line was derived from a peritoneal deposit of a patient diagnosed with papillary cystadenocarcinoma of the ovary (30). The cell line was grown as described previously (29, 31).

### Antibodies and reagents

Polyclonal antibody against phosphorylated (Tyr-705) STAT3 (P-STAT3), total STAT3 (T-STAT3), phosphorylated (Tyr-1007/1008) JAK2 (P-JAK2), and total JAK2 (T-JAK2) were obtained from Cell Signaling Technology (Beverly, MA, USA). Antibodies against cytokeratin 7 (cyt7), Ki67, CA125, E-cadherin, vimentin, CD34, Oct4, and CD117 (c-Kit) used for immunohistochemistry were obtained from Ventana (Roche, AZ, USA). CYT387 [Momelotinib (GS-0387/CYT-0387)] was obtained from Gilead Sciences (CA, USA).

### RNA extraction and quantitative real-time PCR

Tumors obtained from mice were homogenized and cells lysed prior to RNA extractions and cDNA synthesis as described previously (29). For quantitative real-time PCR (q-PCR), four tumors in each group [control, paclitaxel-treated, CYT387-treated, and paclitaxel+CYT387-treated] were analyzed in triplicate as described previously (32). Each gene was validated by using an amplified, purified, and sequenced PCR fragment (originating from HEY cell line) as a positive control. The primers used for 18S, Oct-4A, IL-6, interleukin-6 receptor (IL-6R), glycoprotein 130 (gp130), C-X-C chemokine receptor type 4 (CXCR4), matrix metalloproteinase 2 (MMP-2), and matrix metalloproteinase 9 (MMP-9) are described in Table 1. q-PCR was carried out using ViiA 7 real-time PCR system (Applied Biosystems). Relative gene expression was calculated as 2^−ΔΔ*C_t_*^ using 18S as the endogenous reference gene and the average of the controls as the calibrator.

**Table 1:** **Human oligonucleotide primer sequences for quantitative real-time PCR**.

Gene symbol	Accession no.	Primer sequences from 5′to 3′	Size (bp)
*RNA18S*	NR 003286.2	Forward GTAACCCGTTGAACCCCATT	153
		Reverse CCATCCAATCGGTAGTAGCG	
*POU5F1*	NM 002701.4	Forward CTCCTGGAGGGCCAGGAATC	381
*(OCT4A)*		Reverse CCACATCGGCCTGTGTATAT	
*IL6*	NM 000600.3	Forward TACCCCCAGGAGAAGATTCC	175
		Reverse TTTTCTGCCAGTGCCTCTTT	
*IL6R*	NM 000565.3	Forward CTCCTGCCAGTTAGCAGTCC	198
		Reverse TCTTGCCAGGTGACACTGAG	
*IL6ST*	NM 002184.3	Forward TGTAGATGGCGGTGATGGTA	246
*(gp130)*		Reverse CCCTCAGTACCTGGACCAAA	
*CXCR4*	NM 001008540.1	Forward GAAGCTGTTGGCTGAAAAGG	94
		Reverse CTCACTGACGTTGGCAAAGA	
*MMP2*	NM 004530.4	Forward TTGACGGTAAGGACGGACTC	153
		Reverse ACTTGCAGTACTCCCCATCG	
*MMP9*	NM 004994.2	Forward TTGACAGCGACAAGAAGTGG	179
		Reverse GCCATTCACGTCGTCCTTAT	

### Animal studies

#### Animal ethics statement

This study was carried out in strict accordance with the recommendations in the Guide for the Care and Use of the Laboratory Animals of the National Health and Medical Research Council of Australia. The experimental protocol was approved by the Ludwig/Department of Surgery, Royal Melbourne Hospital, and University of Melbourne’s Animal Ethics Committee (Project-006/11), and was endorsed by the Research and Ethics Committee of Royal Women’s Hospital Melbourne, VIC, Australia.

#### Animal experiments

Female Balb/c *nu/nu* mice (age, 6–8 weeks) were obtained from the Animal Resources Centre, Western Australia. Animals were housed in a standard pathogen-free environment with access to food and water.

HEY cells (5 × 10^6^) were inoculated subcutaneously in each flank of nude mice as described previously (33). Mice were inspected weekly and tumor progression was monitored based on overall health and body weight until one of the pre-determined endpoints was reached. Endpoint criteria included exceeding 20% loss of initial body weight and a general pattern of diminished well-being such as reduced movement due to tumor burden, ulceration of tumors due to constant irritation of the protruding tumors with the mouse bedding, and lethargy resulting from lack of interest in daily activities. As such, the endpoint of the experiment was noted at day 7 (first approach) or day 28 (second approach) after the start of paclitaxel and/or CYT387 treatments. The 7- or 28-day duration was chosen as the tumor volume in the control group reached the defined volume of end point as specified in the Animal Ethics application.

In the first approach, mice were divided into two groups with *n* = 3 mice in each group and HEY cells were inoculated subcutaneously in each flank of nude mice. The first group of mice was treated as a control. After 7 days, the second group of mice was treated once intraperitoneally with 15 mg/kg of body weight of paclitaxel. These mice were followed for 7 days, after which the experiment was terminated.

In the second approach, mice were divided into four groups with *n* = 5 in each group and HEY cells were inoculated subcutaneously in each flank of nude mice. The first group of mice was designated as control. The second group was treated once a week with an intraperitoneal injection of paclitaxel at 15 mg/kg of body weight, 2 days post inoculation of the HEY cells. The third group was treated with the same dose of paclitaxel weekly in addition to daily doses of CYT387 at 5 mg/kg of body weight by oral gavages. The weekly intraperitoneal injection of paclitaxel and oral gavages of CYT387 was continued for 28 days. The fourth group of mice was treated with a daily dose of 5 mg/kg of body weight of CYT387 by oral gavages for 28 days. Mice were euthanized at the experimental endpoint and the tumors were excised for further examination.

Tumor volume measurements were performed with calipers at day 0 and days 7 and 28 (the experimental endpoint). Measurement of tumor volume in cubic millimeter was determined using the formula (length × width^2^)/2; where length was the longest axis and width was the measurement at right angles to the length (33). Fold change in tumor volume was calculated from the ratio of tumor volume at day 0 to day 7 or day 28.

#### Immunohistochemistry

For immunohistochemistry, formalin-fixed, paraffin-embedded 4 μm sections of the xenografts were stained using a Ventana Benchmark Immunostainer (Ventana Medical Systems, Inc., AZ, USA) described previously (29). Detection was performed using Ventana’s ultra view diaminobenzidine (DAB) detection kit (Roche/Ventana, AZ, USA). Tumor sections were dewaxed with Ventana EZ Prep and endogenous peroxidase activity was blocked using the Ventana’s universal DAB inhibitor. Primary antibodies against Oct4, Ki67, cancer antigen 125 (CA125), CD117 (c-Kit), total JAK2 (T-JAK2), phospho-JAK2 (P-JAK2), total STAT3 (T-STAT3), and phospho-STAT3 (P-STAT3) were diluted according to the instruction provided by the manufacturer as described in Table 2. For nuclear staining, the sections were counter stained with Ventana hematoxylin and bluing solution. For each antigen, a parallel paraffin-embedded section was prepared without the primary antibody as a negative control. High-grade serous ovarian tumor sections were used as positive controls.

**Table 2:** **Antibody information**.

Antibodies	Concentrations used	Incubation time (minutes)	Supplier	Catalog number
Mouse anti-human Ki67	2 μg/ml	12	Ventana	790-4286
Mouse anti-human Oct4 (we use Oct3/4)	1.2 μg/ml	32	Novocastra	NCL-L-OCT3/4
Mouse anti-human CD34	0.8 μg/ml	32	Ventana	790-2927
Mouse Anti-Human CD117	5 μg/ml	32	Ventana	790-2951
Rabbit anti-human JAK2	10 μg/ml	40	Cell Signaling	3230
Rabbit anti-human Phospho-JAK2 (Tyr1007/1008) (C80C3)	10 μg/ml	40	Cell Signaling	3776
Mouse anti-human STAT3 (124H6)	2.5 μg/ml	40	Cell Signaling	9139
Mouse anti-human phospho-STAT3 (Tyr705) (E2)	10 μg/ml	40	Cell Signaling	9138
Mouse anti-human CA125	0.23 μg/ml	32	Ventana	760-2610
HRP-conjugated secondary	Unknown, proprietary reagent	8	Ventana	760-500

Immunohistochemistry images were created using an Axioskop 2 microscope, captured using a Nikon DXM1200C digital camera and processed using NIS-elements F3.0 software. Slides were scored independently by four blind reviewers, as described previously (34). For each slide, the extent of positive staining was scored as five grades, namely, 0 (≤10%), 1 (>10–25%), 2 (>25–40%), 3 (≥40–50%), 4 (≥50–75%), and 5 (>75%). The intensity of staining (IS) was classified into four grades: no staining (−), pale brown (1), moderate brown (2) and dark brown (3). Scoring was determined by using the Allred method of visual quantification as per the following formula: percentage of staining (PS) + IS = total score (range 0–8) (35).

### Statistical analysis

Data are presented as mean ± SEM. A probability level of *p* < 0.05 was adopted throughout to determine statistical significance. Treatment groups were compared with the control group using one-way ANOVA followed by Bonferroni or Dunnett’s multiple comparison post-tests.

## Results

### Effect of single dose of paclitaxel on HEY tumors

Subcutaneous injection of mice with HEY cells resulted in the formation of tumors within 5 days. Intraperitoneal injection of paclitaxel (15 mg/kg of body weight) given 7 days post inoculation of HEY cells was well tolerated by the mice. Treatment with a single dose of paclitaxel did not result in any significant change in the tumor volume compared to the control untreated mice (Figures S1A–C in Supplementary Material).

Mouse tumors were excised and assessed by immunohistochemistry. Xenografts that received paclitaxel treatment demonstrated significantly enhanced staining for the CSC-like marker CD117 and the embryonic stem cell maker Oct4 compared to the control group (Figure S2 in Supplementary Material). These results coincided with a significantly enhanced staining for CA125 in the mice treated with paclitaxel compared to the control group (Figure S2 in Supplementary Material). The expression of CD117 and CA125 in untreated and paclitaxel-treated groups was confined to the cytoplasm and cell membranes. The staining of Oct4 was observed in the cytoplasm as well as in the nucleus in both control and paclitaxel-treated mouse tumors (Figure S2 in Supplementary Material).

### Volume of xenografts generated through administration of a combination of daily oral gavages of CYT387 with weekly intraperitoneal paclitaxel injections

**Figure 1:**
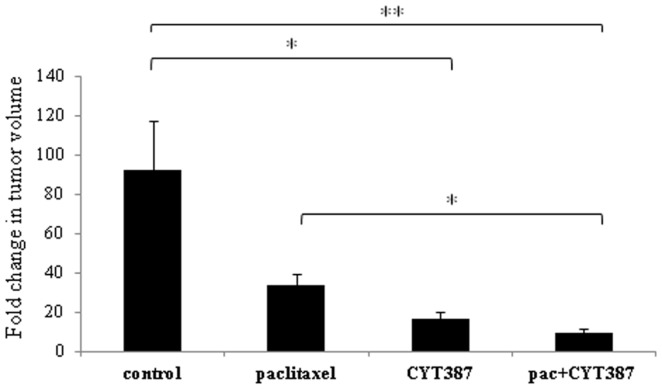
**HEY xenograft volume in mice administered with or without paclitaxel, CYT387 or a combination of paclitaxel and CYT387 (pac + CYT387)**. Average fold change in subcutaneous tumor volume. Tumors were measured at day 0 (prior to treatment) and at the end of the experiment (day 28). Data have been obtained from *n* = 5 mice in each group. Significant intergroup variations are indicated by *p < 0.05, **p < 0.01.

In the second approach, mice were treated with either paclitaxel weekly, or CYT387 daily or treated with a combination of both or observed without intervention (control group) 2 days after subcutaneous inoculation of HEY cells in both flanks of each mouse. Mice were followed for a 28-day treatment period and tumor volumes were analyzed at day 0 (before the start of treatment regimens) versus day 28. A single tumor localized at the site of inoculation (each flank) was obtained from each mouse. Mice in the control group had an approximately 90-fold increase in tumor volume at day 28 compared to day 0 (Figure 1). On the other hand, tumors in mice treated with weekly doses of paclitaxel demonstrated an approximately 30-fold increase in volume at day 28 compared to day 0. However, tumors in mice receiving daily doses of CYT387 demonstrated only an approximately 20-fold increase in tumor volume at day 28 compared to day 0. Remarkably, mice that were treated with a combination of weekly paclitaxel and daily CYT387 developed significantly smaller tumors when compared to paclitaxel-treated and control groups, with a tumor fold change of less than 10-fold at day 28 compared to day 0 (Figure 1). This group had the smallest tumor volume of all the groups. The volume of the tumors produced in the CYT387 treatment group was not significantly different than the paclitaxel treatment group.

### Phenotype of xenografts generated through weekly systemic paclitaxel and daily oral gavages of CYT387

**Figure 2:**
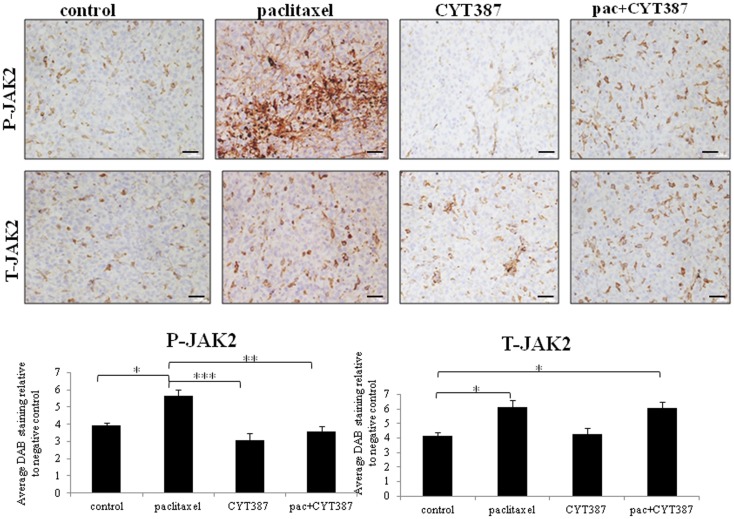
**Immunohistochemistry images of phosphorylated P-JAK2 and T-JAK2 staining in xenografts generated from subcutaneous transplantation of HEY cells into mice administered with or without paclitaxel, CYT387, or pac + CYT387**. Tumor sections were stained with antibodies specific for P-JAK2 and T-JAK2 as described in the Section “Materials and Methods.” Magnification 200×, scale bar = 10 μM. Average DAB intensity and proportion of staining of P-JAK2 or T-JAK2 in xenografts was standardized to a negative control. The quantification was derived from the staining of five independent xenografts in each group. Significant intergroup variations are indicted by *p < 0.05, **p < 0.01, and ***p < 0.001.

**Figure 3:**
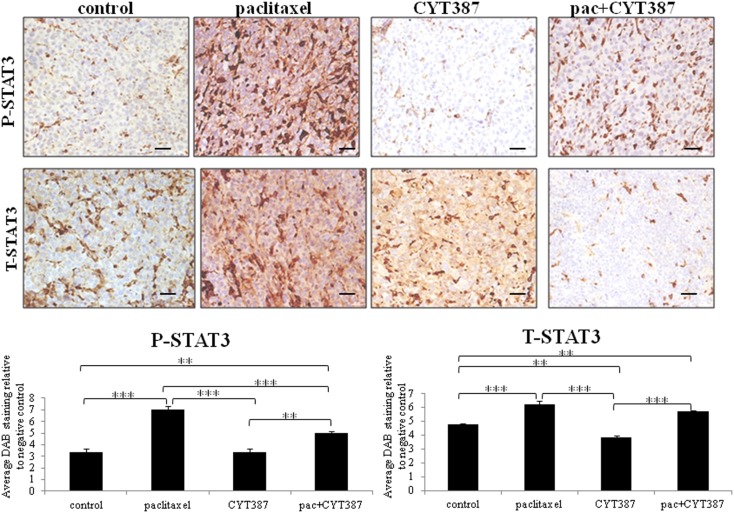
**Immunohistochemistry images of P-STAT3 and T-STAT3 staining in xenografts generated from subcutaneous transplantation of HEY cells into mice administered with or without paclitaxel, CYT387, or pac + CYT387**. Tumor sections were stained with antibodies specific for P-STAT3 and T-STAT3 as described in the Section “Materials and Methods.” Magnification 200×, scale bar = 10 μM. Average DAB intensity and proportion of staining of P-STAT3 or T-STAT3 in xenografts was standardized to a negative control. The quantification was derived from the staining of five independent xenografts in each group. Significant intergroup variations are indicted by **p < 0.01 and ***p < 0.001.

Xenografts were collected and analyzed using immunohistochemistry. The expression of phosphorylated (P) and total (T)-JAK2 and STAT3 was mostly confined to the cytoplasm. However, some scattered nuclear staining was also evident. Staining for phosphorylated (P) and total (T) JAK2 and STAT3 was significantly increased in tumors derived from mice receiving weekly paclitaxel treatment compared to the control group (Figures 2 and 3). Daily administration of CYT387 on its own had no effect on the phosphorylation of JAK2 or STAT3 compared to the control group, however it significantly decreased the endogenous expression of T-STAT3 without any effect on the expression of T-JAK2. Administration of daily CYT387 in conjunction with paclitaxel treatment resulted in tumors that displayed a significantly decreased staining of P-JAK2 and P-STAT3, compared to paclitaxel only treated group. However, no effect on T-JAK2 or T-STAT3 in the paclitaxel+CYT387 group compared to the paclitaxel only treated group could be observed. This suggests that daily doses of CYT387 abolished the paclitaxel-induced activation of the JAK2/STAT3 pathway without having a significant effect on the expression of the total proteins (Figures 2 and 3).

**Figure 4:**
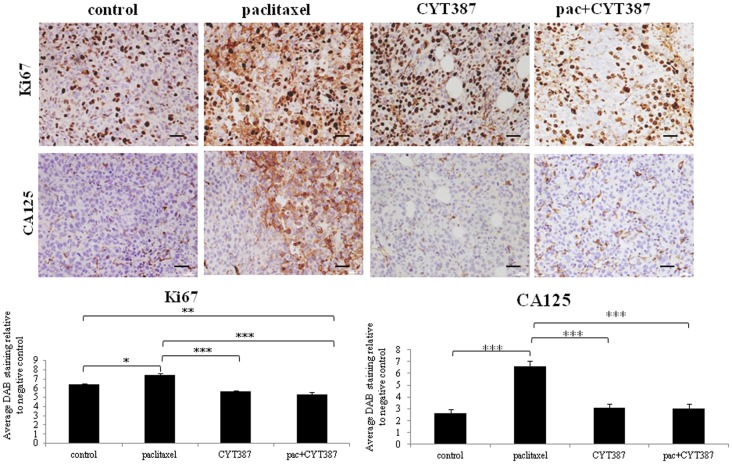
**Immunohistochemistry images of Ki67 and CA125 staining in xenografts generated from subcutaneous transplantation of HEY cells into mice administered with or without paclitaxel, CYT387, or pac + CYT387**. Tumor sections were stained with antibodies specific for Ki67 and CA125 as described in the Section “Materials and Methods.” Magnification 200×, scale bar = 10 μM. Average DAB intensity and proportion of staining of Ki67 or CA125 in xenografts was standardized to negative control. The experiments were performed using five independent samples in each group. Significant intergroup variations are indicted by *p < 0.05, **p < 0.01, and ***p < 0.001.

Mice receiving paclitaxel developed tumors that displayed significantly enhanced staining of the cell proliferation maker Ki67 when compared to the control group (Figure 4). The staining of Ki67 both in the control and treated groups was confined to the nucleus. However, this enhanced nuclear staining of Ki67 in response to paclitaxel administration was significantly reduced when CYT387 was added in combination with paclitaxel (Figure 4). Moreover, mice that received daily CYT387 alone developed tumors that displayed a significantly reduced Ki67 staining when compared to the paclitaxel only treatment group. However, it had no effect on basal Ki67 staining (Figure 4).

**Figure 5:**
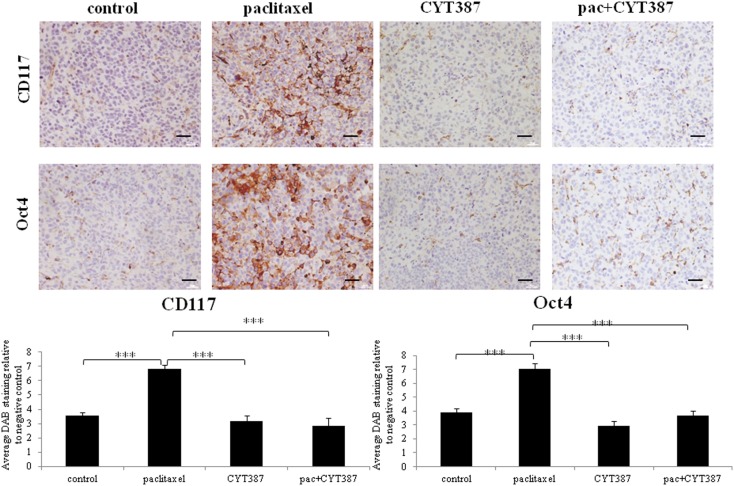
**Immunohistochemistry images of CD117 and Oct4 staining in xenografts generated from subcutaneous transplantation of HEY cells into mice administered with or without paclitaxel, CYT387, or pac + CYT387**. Tumor sections were stained with antibodies specific for CD117 and Oct4 as described in the Section “Materials and Methods.” Magnification 200×, scale bar = 10 μM. Average DAB intensity and proportion of staining of CD117 or Oct4 in xenografts was standardized to a negative control. The experiments were performed using five independent samples in each group. Significant intergroup variations are indicted by ***p < 0.001.

Similar to that demonstrated in the first approach, tumors derived from mice treated with paclitaxel alone displayed significantly enhanced staining for CA125, the CSC-like marker CD117, and the embryonic stem cell marker Oct4, when compared to the control group (Figures 4 and 5). However, tumors derived from mice treated with a combination of paclitaxel and CYT387 demonstrated significantly reduced staining of CD117, Oct4, and CA125 compared to the paclitaxel-treated group (Figures 4 and 5). CYT387 on its own had no effect on the basal expression of CA125, CD117, and Oct4. These results suggest that the addition of CYT387 abrogates the paclitaxel-induced CA125, CD117, and Oct4 expression. The expression of CD117, Oct4, and CA125 was present mostly in the cytoplasm and cell–cell membrane junctions. Very little nuclear staining of Oct4 was also evident in xenografts generated upon paclitaxel treatment.

**Figure 6:**
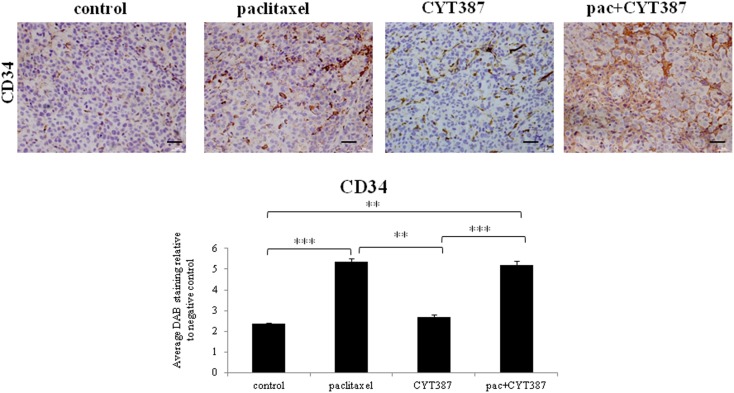
**Immunohistochemistry images of CD34 staining in mice xenografts from subcutaneous transplantation of HEY cells into mice administered with or without paclitaxel, CYT387, or pac + CYT387**. Tumor sections were stained with antibodies specific for CD34 as described in the Section “Materials and Methods.” Magnification 200×, scale bar = 10 μM. Average DAB intensity and proportion of staining of CD34 in xenografts was standardized to a negative control. The experiments were performed using five independent samples in each group. Significant intergroup variations are indicted by **p < 0.01 and ***p < 0.001.

Mice treated with paclitaxel developed tumors with significantly enhanced expression of CD34^+^ cells when compared to the control as well as the CYT387 treatment groups (Figure 6). The addition of CYT387 to paclitaxel did not reduce the paclitaxel-induced enhanced expression of CD34 which was mostly cytoplasmic.

**Figure 7:**
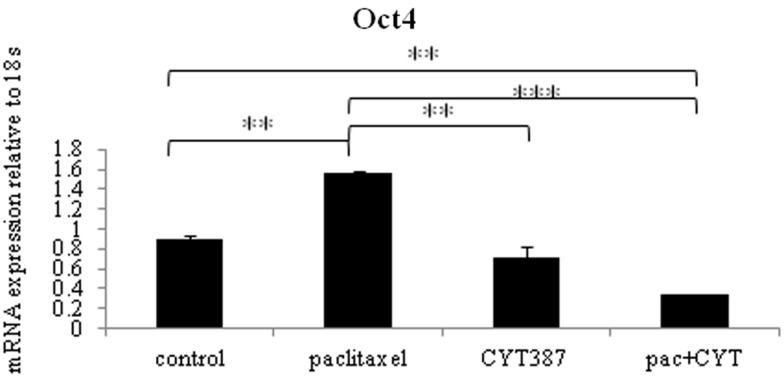
**The mRNA expression of embryonic stem cell marker Oct4 in xenografts generated from subcutaneous transplantation of HEY cells into mice administered with or without paclitaxel, CYT387, or a combination of paclitaxel and CYT387 (pac + CYT)**. mRNA from xenografts generated from the control group and treatment groups was extracted, cDNA was prepared, and q-PCR for Oct4 was performed as described in the Section “Material and Methods.” The resultant mRNA levels were normalized to 18S mRNA. The experiments were performed using five independent samples in triplicate. Significant intergroup variations are indicated by **p < 0.01 and***p < 0.001.

To determine if changes in the embryonic stem cell marker Oct4 seen at the protein level in mouse xenografts were consistent at the mRNA level, q-PCR on cDNA prepared from RNA extracted from mouse tumors was performed. An analysis of the embryonic stem cell marker Oct4 revealed significantly enhanced mRNA expression in tumors derived from mice treated with paclitaxel when compared to the control group (Figure 7). Consistent with the Oct4 immunohistochemistry staining, the addition of daily CYT387 treatment resulted in a significant reduction of Oct4 mRNA in tumors compared to mice treated with paclitaxel (Figure 7). Treatment with CYT387 alone did not result in any significant change in the mRNA expression of Oct4 compared to the control untreated group (Figure 7).

### Effect of oral gavages of CYT387 in combination with systemic administration of paclitaxel on interleukin-6-mediated responses in mouse xenografts

**Figure 8:**
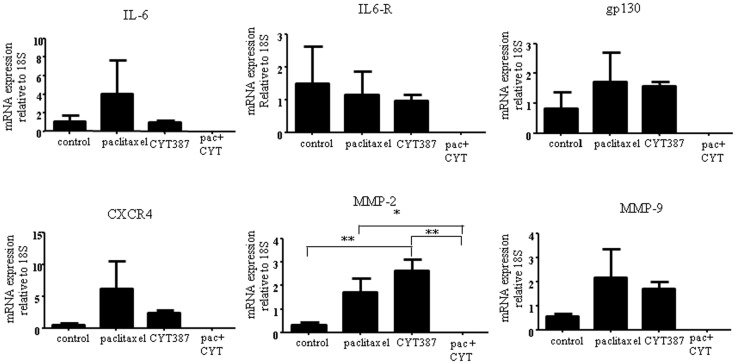
**The mRNA expression of IL-6, IL-6R, gp130, CXCR4, MMP-2, and MMP-9 in xenografts generated from subcutaneous transplantation of HEY cells into mice administered with or without paclitaxel, CYT387, or pac + CYT**. mRNA from xenografts generated from the control group and treatment groups was extracted, cDNA was prepared, and q-PCR for IL-6, IL-6R, gp130, MMP-2, MMP-9, and CXCR4 was performed as described in the Section “Material and Methods.” The resultant mRNA levels were normalized to 18S mRNA. The experiments were performed using four independent samples in triplicate. Significant intergroup variations are indicated by *p < 0.05 and **p < 0.01.

We investigated whether the mRNA expression of IL-6 in the control tumor xenografts had any correlation with the mRNA levels of invasion-associated genes such as CXCR4, MMP-2, and MMP-9 in response to systemic administration of paclitaxel and oral gavages of CYT387. Untreated control and paclitaxel administered mouse tumors expressed human IL-6R as well as gp130 mRNA (Figure 8). There were increased trends in the expression of IL-6, CXCR4, MMP-2, and MMP-9 in the paclitaxel-treated group compared to the control group. This increased trend however, did not receive statistical significance between the two groups (Figure 8). When the combination of paclitaxel and CYT387 was administered, the mRNA expression of IL-6R, gp130, IL-6, CXCR4, MMP-2, and MMP-9 did not change relative to the house keeping gene 18S (Figure 8). On the other hand, CYT387 administration on its own significantly increased the mRNA expression of MMP-2 compared to the control group. An increased trend in the expression of MMP-9 in the CYT387 treatment group compared to control was also observed but it did not receive statistical significance (Figure 8). No change in the mRNA expression of 18S was observed under the same conditions (Figure S3 in Supplementary Material).

## Discussion

CYT387 is an orally available, potent small molecule inhibitor of the JAK1/2 pathway currently undergoing Phase I/II clinical trials for the treatment of myelofibrosis, a frequently diagnosed fatal myeloproliferative disorder (36). CYT387 has so far been the best candidate among the JAK inhibitors for the management of myelofibrosis with the preliminary data showing significant responses with a low level of toxicity (http://www.gilead.com/research/pipeline). CYT387 has demonstrated efficacy in a JAK2V617F mutation-associated animal model where it inhibited STAT3 functions associated with constitutively activated JAK2, by normalizing inflammatory cytokines (37). In the murine JAK2V617F mutation-associated animal model, CYT387 also normalized white cell counts, the hematocrit, and also restored the normal spleen size (37). Preclinical analysis has shown CYT387 to be well tolerated in mice when administered orally at doses up to 50 mg/kg of body weight, with no sign of overt toxicity (37). Besides myeloproliferative disorders, CYT387 has the potential for the treatment of solid and hematological malignancies and inflammatory conditions (36). In this proof of principle study, we demonstrate the novel effect of CYT387 in combination with paclitaxel in significantly suppressing the tumor growth greater than that achieved by paclitaxel on its own. We also show suppression of the expression of CA125, Oct4, and CD117 by CYT387, induced by the activation of JAK2/STAT3 pathway in response to systemic paclitaxel administration in an ovarian xenograft model.

We have recently demonstrated that human ovarian cancer cell lines as well as primary- and ascites-derived ovarian cancer cells treated with cisplatin or paclitaxel generate a surviving residual population of cells which display enhanced expression of the chemoresistant-associated markers ERCC1 and β-tubulin as well as enhanced expression of CSC-like markers CD44, CD24, CD133, CD117, and EpCAM, compared to parental untreated ovarian cancer cells (6, 9, 29). In addition, xenotransplantation studies showed that chemotherapy-treated ovarian cancer cells generate significantly larger tumor burden compared to untreated cells and retain an enhanced stemness profile (29). This suggests that some CSC-like and chemoresistant characteristics may be synchronously regulated in the residual cells that survived chemotherapy (7). In this study, we provide further novel data which demonstrates that Oct4 and CD117 expression are enhanced in a mouse xenograft model by intraperitoneal administration of chemotherapy (paclitaxel) after the subcutaneous implantation of an ovarian cancer cell line. We also demonstrate that the expression of Oct4 and CD117 in tumors generated in response to multiple doses of chemotherapy can be suppressed by the administration of a novel small molecule JAK2-specific inhibitor CYT387. The advantage of using a subcutaneous instead of the intraperitoneal model of ovarian tumor is that it allows for the accurate measurement of tumor volume, thus permitting the monitoring of tumor growth in response to treatments.

In the first part of this study, we demonstrate that a single systemic administration of paclitaxel 1 week after subcutaneous implantation of ovarian cancer cells led to a tumor which had a significant enhancement in the expression of Oct4 and CD117 within the 7 days post treatment. This enhancement in the Oct4 and CD117 expression coincided with a significant enhancement of CA125 staining. Such dramatic changes in Oct4, CD117, and CA125 staining had no bearing on tumor volume within the 7 days after paclitaxel administration. This suggests that although no reduction in tumor volume was observed, a single dose of paclitaxel treatment had imposed certain molecular changes in the paclitaxel surviving residual tumor populations. This process likely occurs consecutively in chemoresistant populations while the chemosensitive populations undergo cell death in response to paclitaxel treatment, a process that results in the eradication of the majority of the tumor mass leaving behind theoretically the CSC-enriched tumor initiating residual disease (38).

In the second approach, we demonstrate that weekly treatment of paclitaxel over 28 days resulted in a dramatic reduction of the mouse tumor volume as evidenced by a 30-fold increase in the tumor volume in mice treated with paclitaxel when compared to a ~90-fold increase in the volume in mice not receiving the treatment. These results suggest that systemic weekly administration of paclitaxel-induced cytotoxic and anti-proliferative effects on the tumor population restricting the growth of the tumors compared to the control untreated tumors. However, CYT387 in combination with paclitaxel was able to significantly reduce the tumor volume greater than that can be achieved with paclitaxel alone. This was achieved by using a concentration of CYT387 (5 mg/kg of body weight) that was one-tenth of that used in the JAK2V617F mutation-associated animal model (50 mg/kg of body weight) (37). In addition, CYT387 was administered twice a day in the JAK2V617F mutation-associated animal model compared to a single administration of the drug each day in our study. Hence, it can be anticipated that increasing the concentration of CYT387 toward the same level as that used for the JAK2V617F mutation-associated animal model would be well tolerated in our mouse model and therefore has the potential of reducing tumor volume further.

Tumor cells from mice which survived the paclitaxel treatment were found to have an activated JAK2/STAT3 pathway and to have significantly enhanced staining of embryonic stem cell transcription factor Oct4 and CSC-like marker CD117. In addition, tumors derived from mice treated with paclitaxel showed significantly enhanced CA125 staining. These novel findings suggest that while the tumor volume was smaller in paclitaxel-treated mice, these tumor cells underwent specific molecular changes. As elevated level of CA125 is the hallmark of ovarian cancer diagnosis and frequently observed in recurrent disease, enhanced expression of CA125 in paclitaxel-treated tumor cells may suggest priming of the residual cells for recurrence.

The above *in vivo* mice data are consistent with the data obtained after analyzing several stem cell markers in ovarian tumor specimens collected at diagnosis (before treatment), after chemotherapy treatment and at first recurrence (10). It has been reported that CD133, CD44, and ALDH1A1 were present at low numbers in primary tumors, however, this was found to increase in tumor specimens taken immediately after chemotherapy treatment and then reduced to initial numbers in recurrent tumors, suggesting that these so-called “CSC markers” identify “chemoresistant cells.” Such observations in animal models and clinical specimens suggest that chemotherapy treatment may induce a “chemoresistant niche,” which protects residual chemoresistant cells from cell death by promoting a microenvironment appropriate for the survival of CSCs. In that context, CD133 positive colon CSCs have been shown to protect themselves *in vivo* from apoptosis by autocrine secretion of interleukin-4 (IL-4) (39). Paclitaxel treatment has been shown to promote angiogenesis in tumors through the mobilization of bone marrow-derived endothelial cells to tumors by an acute drug-mediated release of stromal-derived factor-1 (SDF-1) and G-CSF (40).

We also demonstrate that combining a daily dose of CYT387 with weekly paclitaxel treatment resulted in the development of mouse tumors which had a significantly reduced activation of the JAK2/STAT3 pathway compared to the group which received only paclitaxel. This correlated with the significantly reduced expression of the paclitaxel-induced Oct4, CD117, CA125, and Ki67 expression. In addition, the tumor volume in the mice group that received daily doses of CYT387 in combination with weekly paclitaxel treatment was significantly smaller compared to the treatment group that received only paclitaxel.

Our data also demonstrate a significantly enhanced accumulation of CD34^+^ cells in tumors treated with paclitaxel compared to control untreated tumors. CD34^+^ cells are a well-characterized population of mesenchymal stem cells derived from bone marrow or adipose tissue that have been used clinically to reconstitute the hematopoietic system after radiation or chemotherapy (41). More recently, CD34^+^ cells have also been shown to induce therapeutic angiogenesis in animal models of myocardial, peripheral, and cerebral ischemia by direct incorporation of cells into the expanding vasculature and/or paracrine secretion of angiogenic growth factors that supports the developing microvasculature (42). The fact that CYT387 had no effect on the accumulation of CD34^+^ cells in response to paclitaxel treatment indicates that CYT387 may not have an effect on angiogenesis.

We have recently shown enhanced secretion of interleukin-6 (IL-6) and G-CSF and activation of associated downstream STAT3 pathway in several ovarian cancer cell lines in response to cisplatin or paclitaxel treatments *in vitro* (7, 43). This suggests that an “acute” drug-induced secretory response is promoted in the tumor microenvironment following therapeutic administration, which may have a negative impact on the therapeutic response and act in favor of tumor cells by protecting them from the cytotoxic effects of the chemotherapy. In addition, ovarian cancer-related inflammation has recently been shown to be associated with autocrine cytokine network mediated by tumor necrosis factor (TNF), CXCL12 (also known as SDF-1, ligand for CXCR4 receptor), and IL-6 (44). Autocrine secretion of IL-6 by tumor cells or the associated infiltrated cells, not only promotes tumor growth and invasion (45) but also facilitates chemoresistance (43, 46). A recent study has demonstrated metastatic and drug-resistant recurrent ovarian tumors to have a significantly higher IL-6 expression compared to the matched primary tumors. In that study, the use of a monoclonal IL-6 antibody was shown to suppress IL-6 induced STAT3 phosphorylation and nuclear translocation. This resulted in the decreased expression of STAT3 downstream targets such as Mcl-1 and sensitization of paclitaxel-resistant ovarian cancer cell lines to chemotherapy (27). Our study on the other hand, showed no significant increase in human IL-6 mRNA expression and its downstream metastasis-associated genes MMP-2, MMP-9, and CXCR4 (receptor for CXCL12) in mouse tumors generated during systemic administration of paclitaxel. This suggests that the activation of STAT3 observed in the paclitaxel-treated mouse xenografts may have been triggered by stimulatory agent(s) other than IL-6.

The results from this study reflect the poorer outcomes for patients receiving paclitaxel on its own as a first line chemotherapy. However, these results also provide fresh hope for the potential of a new combination therapy involving CYT387. For the first time, we demonstrate that while the tumor volumes are kept small as a result of paclitaxel treatment, populations of tumor cells within the residual tumors retain the activated JAK2/STAT3 pathway and are enriched in markers such as Oct4 and CD117. We propose that treatment of patients by first line chemotherapy is in fact a process that enables chemotherapy surviving cells to undergo molecular activation of the JAK2/STAT3 pathway. Our data suggests that the inhibition of JAK2/STAT3 pathway by CYT387 at a very low concentration in combination with paclitaxel can suppress the molecular changes induced by chemotherapy in the residual tumors. This can result in a smaller tumor volume than that achieved by the chemotherapy alone. These preliminary “proof of concept” data warrant further investigation of CYT387 in preclinical and clinical models. One of the potential positive outcomes of combining CYT387 with the first line chemotherapy may be a longer disease-free survival period or a decreased incidence of recurrence or perhaps even prevention of the inevitable emergence of fatal recurrent disease.

## Authors Contribution

Khalid Abubaker designed the study, performed the experiments, and contributed to the writing of the manuscript; Rodney B. Luwor helped with the animal experiments; Ruth Escalona designed primers and contributed to the PCR experiments; Orla McNally, Michael A. Quinn, Erik W. Thompson, and Jock K. Findlay edited the manuscript; Nuzhat Ahmed conceived the idea, designed the study, and wrote the manuscript.

## Conflict of Interest Statement

The authors declare that the research was conducted in the absence of any commercial or financial relationships that could be construed as a potential conflict of interest.

## Supplementary Material

The Supplementary Material for this article can be found online at http://www.frontiersin.org/Journal/10.3389/fonc.2014.00075/abstract

Figure S1 **Tumor volume in mice treated with a single dose of paclitaxel**. **(A)** Representative image of subcutaneous tumors in control and paclitaxel-treated mice. **(B)** Fold change in tumor volume (mm^3^) at the end of the study (7 days post treatment) was standardized to initial tumor volume prior to receiving paclitaxel treatment. Data were obtained from *n* = 3 mice in each group. No significant difference between treatment groups was observed.

Figure S2 **Immunohistochemistry images of CD117, Oct4, and CA125 staining in HEY xenografts derived from mice treated with or without a single dose of paclitaxel**. Tumor sections were stained with antibodies specific for CD117, Oct4, and CA125 as described in the Section “Materials and Methods.” Average DAB intensity and proportion of staining for CD117, Oct4, and CA125 in mouse tumors was standardized to a negative control. The experiment was performed on three independent xenografts from each group. Significant intergroup variations are indicated by ***p < 0.001. Magnification 200×, scale bar = 10 μM.

Figure S3 **The mRNA expression of housekeeping gene 18S in xenografts generated from subcutaneous transplantation of HEY cells into mice administered with or without paclitaxel, CYT387, or pac + CYT**. mRNA from xenografts generated from the control group and treatment groups was extracted, cDNA was prepared, and q-PCR for 18S was performed as described in the Section “Materials and Methods.” The experiments were performed using four independent samples in triplicate.
