# Supplementary figures and images for: Targeted Disruption of the JAK2/STAT3 Pathway in Combination with Systemic Administration of Paclitaxel Inhibits the Priming of Ovarian Cancer Stem Cells Leading to a Reduced Tumor Burden
Source: Front Oncol. 2014 Apr 9;4:75. doi: 10.3389/fonc.2014.00075 (PMC3988380; doi:10.3389/fonc.2014.00075)

## Supplementary Fig 1

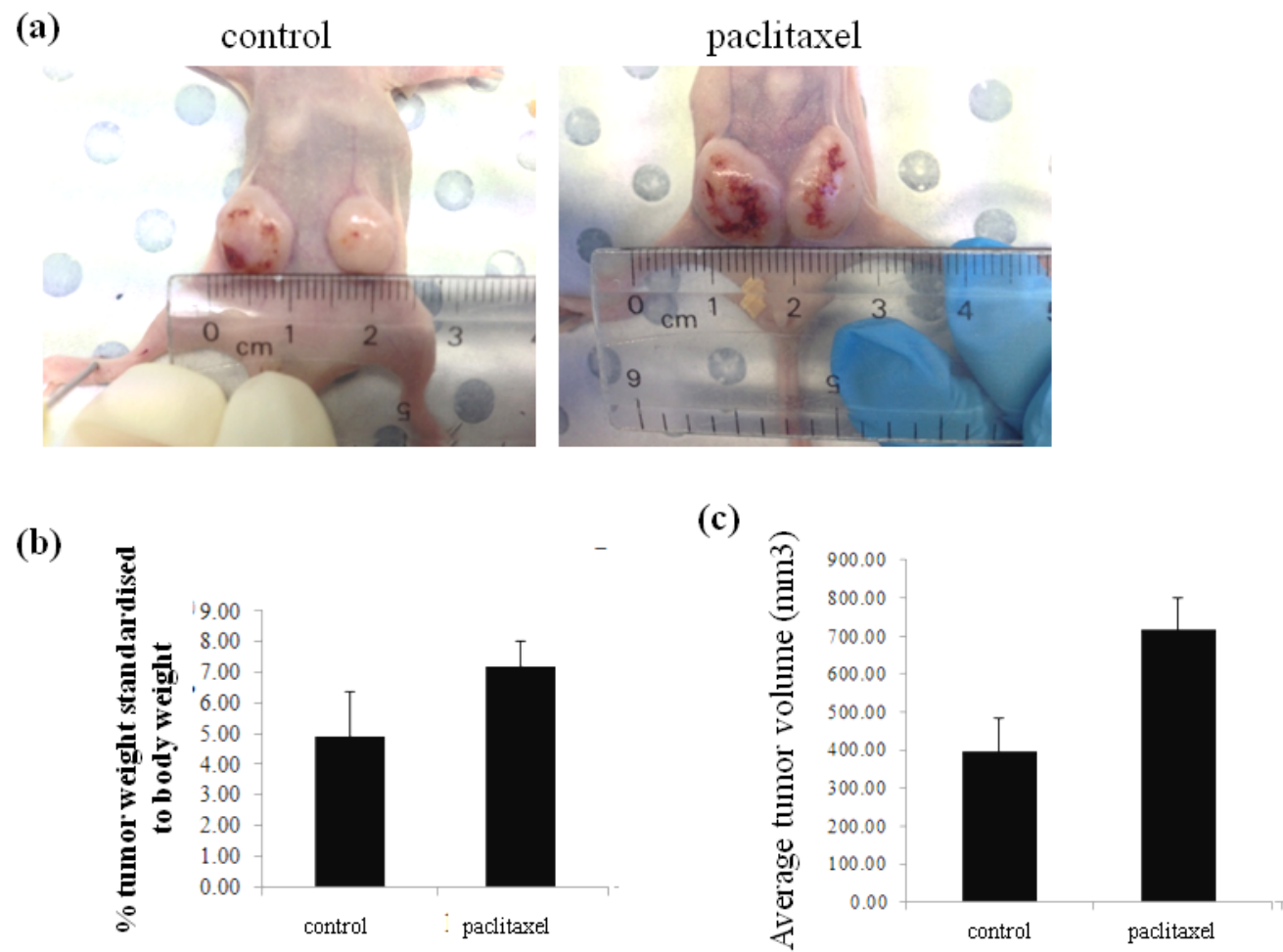

**Supplementary Figure 2**

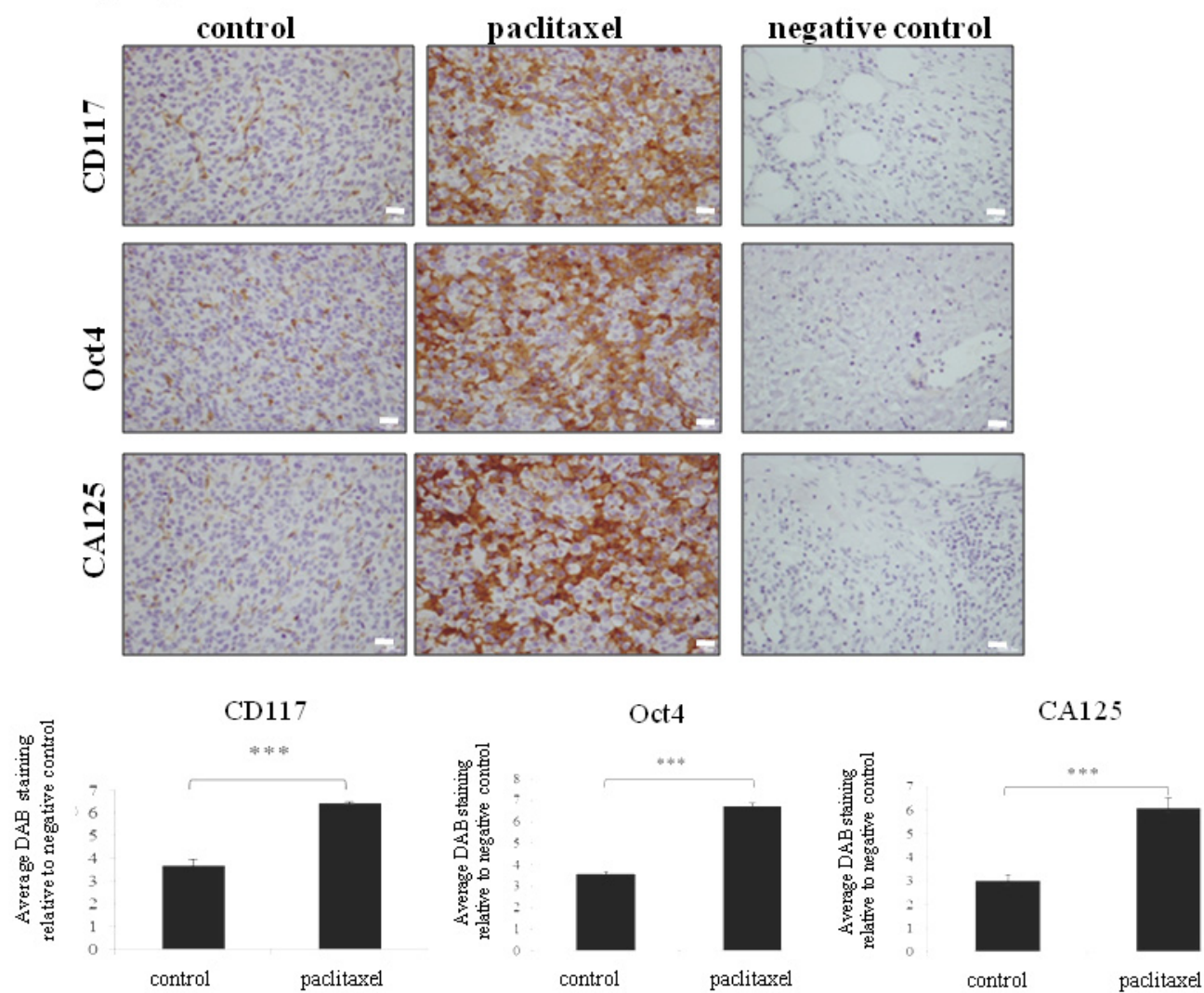

**Supplementary  
Figure 3**

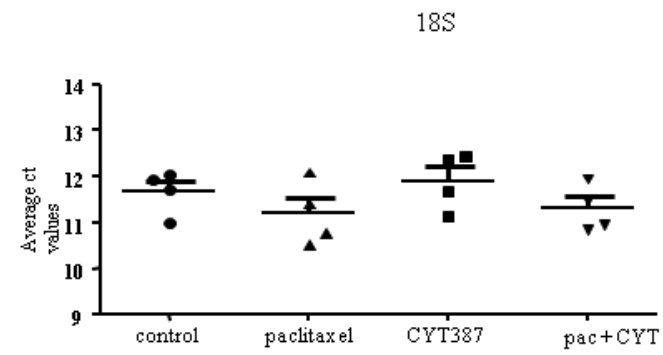

Supplement: Figure S1 — Tumor volume in mice treated with a single dose of paclitaxel. (A) Representative image of subcutaneous tumors in control and paclitaxel-treated mice. (B) Fold change in tumor volume (mm3) at the end of the study (7 days post treatment) was standardized to initial tumor volume prior to receiving paclitaxel treatment. Data were obtained from n = 3 mice in each group. No significant difference between treatment groups was observed. [file Presentation1.PDF]
